# Ser1333 phosphorylation indicates ROCKI activation

**DOI:** 10.1186/1423-0127-20-83

**Published:** 2013-10-29

**Authors:** Hsiang-Hao Chuang, Shao-Wei Liang, Zee-Fen Chang, Hsiao-Hui Lee

**Affiliations:** 1 Institute of Biochemistry and Molecular Biology, National Yang-Ming University, No. 155, Sec. 2, Linong St, Taipei 11221, Taiwan; 2 Department of Life Sciences and Institute of Genome Sciences, National Yang-Ming University, No. 155, Sec. 2, Linong St, Taipei 11221, Taiwan

**Keywords:** Rho-associated protein kinase (ROCK), RhoA, Marker

## Abstract

**Background:**

Two isoforms of Rho-associated protein kinase (ROCK), ROCKI and ROCKII, play a pivotal role in regulation of cytoskeleton and are involved in multiple cellular processes in mammalian cells. Knockout mice experiments have indicated that the functions of ROCKI and II are probably non-redundant in physiology. However, it is difficult to differentiate the activation status of ROCKI and ROCKII in biological samples. Previously, we have identified phosphorylation site of ROCKII at Ser1366 residue sensitive to ROCK inhibition. We further investigated the activity-dependent phosphorylation site in ROCKI to establish the reagents that can be used to detect their individual activation.

**Results:**

The phosphorylation site of ROCKI sensitive to its inhibition was identified to be the Ser1333 residue. The ROCKI pSer1333-specific antibody does not cross-react with phosphorylated ROCKII. The extent of S1333 phosphorylation of ROCKI correlates with myosin II light chain phosphorylation in cells in response to RhoA stimulation.

**Conclusions:**

Active ROCKI is phosphorylated at Ser1333 site. Antibodies that recognize phospho-Ser1333 of ROCKI and phospho-S1366 residues of ROCKII offer a means to discriminate their individual active status in cells and tissues.

## Background

Two isoforms of Rho-associated protein kinase (ROCK), ROCKI (also called ROKβ) and ROCKII (also known as Rho kinase and ROKα) have been identified as RhoA-GTP interacting proteins in mammals [1,2]. They are serine/threonine kinases important for regulation of actin dynamics and cytoskeleton organization [3-5]. These two human kinases share 64% homology in amino acid sequence with 89% identity in the catalytic kinase domain [5]. They contain a Rho-binding domain (RBD) in the coiled-coil region and a pleckstrin homology (PH) domain in the C-terminal region, which folds back onto the N-terminal kinase domain to autoinhibit kinase functions. GTP-RhoA binding relieves the auto-inhibition, switching-on the kinase activity [6]. ROCKI and ROCKII have common substrates, such as myosin light chain (MLC), myosin binding subunit (MYPT1) of the MLC phosphatase, LIM kinases (LIMK1 and LIMK2), α-adducin, ezrin-radixin-moesin (ERM) proteins, and etc. [4,5,7-9]. Collectively, the kinase activation promotes the stabilization of actin filaments and myosin activity to increase actomyosin-driven cellular contractility [10,11]. In addition to regulation by RhoA binding, ROCKs are negatively regulated by distinct binding proteins or phosphorylation. For example, Gem and RhoE specifically inhibit ROCKI and Rad for ROCKII inhibition [5,12]. ROCKII has been shown to be phosphorylated at Tyr722 residue by Src kinase to decrease its affinity to GTP-RhoA [13], and dephosphorylated by Shp2 phosphatase [14]. Therefore, the activity of ROCKI and II *in vivo* could be highly dependent on the cellular context.

To know the distinct biological roles of ROCKI and ROCKII, the ROCKI^−/−^ and ROCKII^−/−^ mice have been generated [15,16]. ROCKI^−/−^ mice are postnatal lethal, because of impairment of umbilical ring closure [16], and ROCKII^−/−^ mice are embryonic lethal at the percentage of 90% due to the dysfunction of placenta and intrauterine growth retardation caused by thrombus formation in the labyrinth layer of placenta [15]. These studies suggest that ROCKI and ROCKII have distinct functions in development.

Many reports have highlighted the association of ROCK activation with cancer progression and suggest the potential of ROCK as therapeutic targets in cancer [17-19]. The level of ROCKI RNA in tumor tissue correlates with the tumor grade and poor overall survival in breast cancer patients [20], and higher level of ROCKI protein has been found in osteosarcoma tissues [21]. As for ROCKII, higher expression has been reported in aggressive hepatocellular carcinomas, colon and bladder cancers [22-24]. Considering that the expression level at mRNA or protein of ROCK may not be necessarily correlated with their kinase activity, we developed the reagents that can directly and specifically detect the activation status of ROCKI and ROCKII in cells and tissues by identification of their corresponding phosphorylation sites. Our previous results have provided evidence that ROCKII at Ser1366 residue reflects its kinase activation [25]. In this study, we further showed activated ROCKI with phosphorylation at Ser1333 residue. Thus, the specific antibodies, one against ROCKI Ser1333 phosphorylation and another against ROCKII Ser1366 phosphorylation, can be used to detect the active form of ROCKI and ROCKII, respectively.

## Methods

### Plasmids and reagents

The S1333A mutation of ROCKI was introduced to wild-type pCMV2-flag-ROCKI described previously [25] using the Quick-Change site-directed mutagenesis kit (Stratagene). Y27632 was from Calbiochem-Novabiochem Corp.; λPPase was from New England Biolabs; nocodazole, anti-flag and anti-MLC antibodies were from Sigma-Aldrich; anti-ROCKI, anti-ROCKII and anti-RhoA antibodies were purchased from Santa Cruz Biotechnology; anti-phospho-MLC2 (T18/S19) antibody from Cell Signaling Technology; anti-pSer1366 ROCKII antibody was described previously [25].

### Cell culture and transient transfection

Normal mouse embryonic fibroblasts (MEFs) and HEK293T cells were maintained in Dulbecco’s modified Eagle’s medium (DMEM) supplemented with 10% (v/v) fetal bovine serum (FBS) in a humidified atmosphere of 5% CO_2_/95% air at 37°C. For transient transfection experiments, HEK293T cells were transfected by PolyJet reagent (SignaGen Laboratories).

### Immunoprecipitation and *in vitro* kinase reaction

Flag-ROCKI-expressing cells were harvested in an IP buffer (1% NP-40, 5% glycerol, 50 mM Tris–HCl, pH 7.4, 150 mM NaCl, 1 mM PMSF, 50 mM NaF, 2 mM Na_3_VO_4_ and protease inhibitor cocktail). The lysates after pre-clearance were incubated with anti-flag antibody conjugated agarose beads (Sigma-Aldrich) at 4°C for 1 hr. The immunoprecipitates were pre-incubated with or without 100 μM of Y27632, which was followed by incubation with a kinase buffer (50 mM Tris–HCl, pH7.4, 10 mM MgCl_2_, 1 mM EGTA, 0.5 mM DTT, 5 mM NaF, 0.1 mM Na_3_VO_4_, and 20 μM ATP) containing 5 μCi of [γ-^32^p]ATP at 30°C for 20 min. The reaction was stopped and products were separated by SDS-PAGE, transferred to a PVDF membrane. The phosphorylation status and amounts of the proteins were detected by autoradiography and Western blotting with anti-ROCKI antibody, respectively.

### Phospho-specific antibody generation

The polyclonal anti-pS1333 ROCKI antibody was raised using phosphopeptide containing phosphorylated Ser1333 of ROCKI conjugated with keyhole limpet haemocyanin (KLH) as an antigen to immunize rabbits. Anti-sera were collected and sequentially affinity purified by phosphopeptide- and non-phosphopeptide-conjugated columns (ICON Biotechnology Co., Itd., Taiwan).

## Results and discussion

### Identification of activity-dependent phosphorylation site of ROCKI

**Figure 1:**
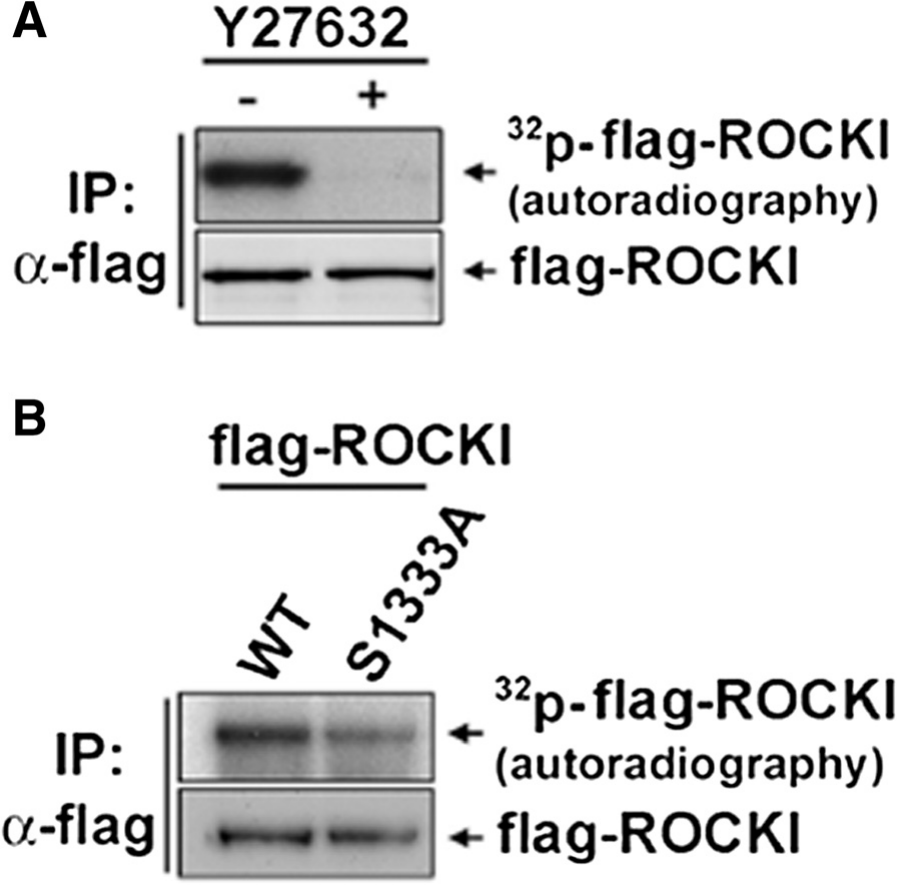
**ROCKI is phosphorylated at Ser1333 residue. (A)** The flag-ROCKI protein was expressed in HEK293T cells and immunoprecipitated with anti-flag antibody. Equal amounts of immunoprecipitated complex were pre-incubated with 100 μM of Y27632 for 20 min and subjected to an *in vitro* kinase reaction with 5 μCi of [γ-^32^P] ATP. **(B)** Wild-type (WT) and S1333A mutant of flag-ROCKI proteins were immunoprecipitated for *in vitro* kinase reaction. After 20 min, the reaction was stopped and proteins were then applied to SDS-PAGE followed by autoradiography. The protein level of flag-ROCKI was determined by Western blotting with anti-ROCKI antibody.

To search for kinase-dependent phosphorylation site of ROCKI, we performed an *in vitro* kinase reaction using immunoprecipitated flag-tagged ROCKI protein in the presence of [γ-^32^P]ATP. Autoradiography detected the phosphorylation signal, which was abolished by including ROCK inhibitor Y27632 in the *in vitro* kinase reaction (Figure 1A). Ser1333 residue in human ROCKI sequence is corresponding to the Ser1366 of ROCKII and is conserved in vertebrates. We then isolated immunocomplex of wild-type (WT) and S1333A mutant of flag-ROCKI proteins in cells for the *in vitro* kinase assay using [γ-^32^P]ATP labeling. The result showed that S1333A mutation markedly reduced the intensity of [γ-^32^P]ATP labeling in ROCKI (Figure 1B), indicating that Ser1333 residue is one of the phosphorylation sites of ROCKI dependent on its own kinase activity.

### Validation of Ser1333 phosphorylation of ROCKI by specific antibody

**Figure 2:**
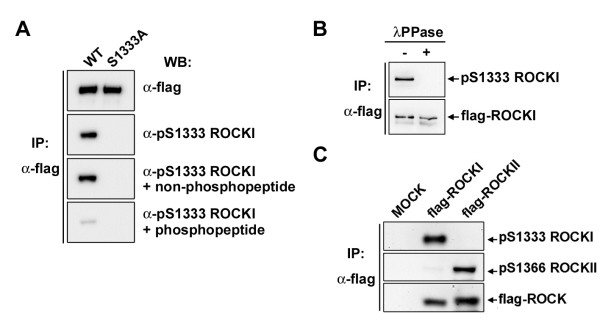
**Validation of anti-pSer1333 ROCKI antibody. (A)** Wild-type (WT) or S1333A mutant of flag-ROCKI proteins were expressed in HEK293T cells and immunoprecipitated with anti-flag antibody followed by probed with anti-flag or anti-pSer1333 ROCKI antibody with or without peptide competition in a same set of sample. **(B)** WT flag-ROCKI immunoprecipitates were incubated with or without λPPase at 30°C for 20 min followed by Western blot analysis as indicated. **(C)** Flag-tagged ROCKI and ROCKII proteins were immunoprecipitated for Western blot analysis using anti-pSer1333 ROCKI, anti-pSer1366 ROCKII, and anti-flag antibodies in a same set of sample.

To assure that phosphorylation at Ser1333 is a mark of ROCKI activation in biological samples, we then generated a phospho-specific antibody by a phosphopeptide containing pSer1333 of ROCKI. The specificity of this antibody was tested by Western blot analysis of the immunoprecipitated WT and S1333A mutant of flag-ROCKI proteins. The results showed that purified anti-pS1333 ROCKI antibody was capable of detecting the phosphorylation of immunoprecipitated WT but not S1333A flag-ROCKI protein. The signal was neutralized by phosphorylated peptide but not by non-phosphorylated peptide (Figure 2A). Treatment of flag-ROCKI (WT) immunoprecipitates with λ protein phosphatase (λPPase) abolished the signal (Figure 2B). These data indicate the specificity of anti-pSer1333 ROCKI antibody. Given the similarity in amino acid sequence surrounding Ser1333 in ROCKI and Ser1366 in ROCKII, we used flag-tagged ROCKI and ROCKII immunoprecipitates to verify the specificity of these two antibodies. As shown in Figure 2C, neither did anti-pSer1333 ROCKI antibody cross-react with ROCKII, nor anti-pSer1366 ROCKII antibody to ROCKI.

### Detection of endogenous ROCKI activation by assessing Ser1333 phosphorylation

**Figure 3:**
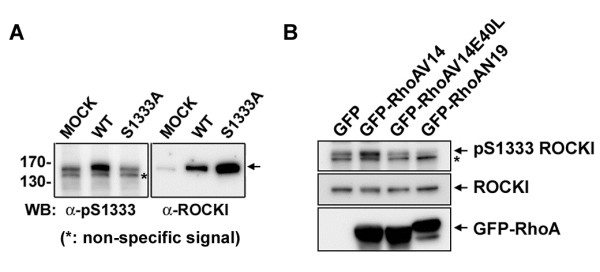
**Detection of endogenous ROCKI phosphorylation at Ser1333 residue. (A)** HEK293T cells expressing WT or S1333A mutant of flag-ROCKI were harvested for Western blot analysis with anti-pS1333 ROCKI and anti-ROCKI antibodies. **(B)** Cells were transfected with the expression constructs of GFP, GFP-RhoAV14, GFP-RhoAV14E40L, or GFP-RhoAN19 and harvested for Western blotting with antibodies as indicated. *, non-specific signal.

We further used this antibody for direct Western blot analysis of lysates from HEK293T cells expressing WT and S1333A ROCKI. As shown in Figure 3A, the antibody detected a major signal in WT ROCKI but not S1333A mutant. A lower band was a non-specific signal because the intensity was similar regardless of the ectopic expression of ROCKI. Next, we assessed the change in Ser1333 phosphorylation of endogenous ROCKI in response to RhoA activation. To this end, HEK293T cells were transfected with the expression construct of GFP-RhoAV14, a constitutively active form, GFP-RhoAN19, a dominant negative form, or GFP-RhoAV14E40L, a constitutive active mutant defective in interaction with ROCK [26], for Western blot analysis. The level of Ser1333 phosphorylation of ROCKI in cells was increased by expression of GFP-RhoAV14. However, expression of GFP-RhoAV14E40L had no effect on the level Ser1333 phosphorylation of ROCKI. Expression of GFP-RhoAN19 reduced ROCKI Ser1333 phosphorylation (Figure 3B). These data suggest that the activation of endogenous ROCKI by RhoA can be specifically detected by Western blot analysis using anti-pSer1333 ROCKI antibody.

**Figure 4:**
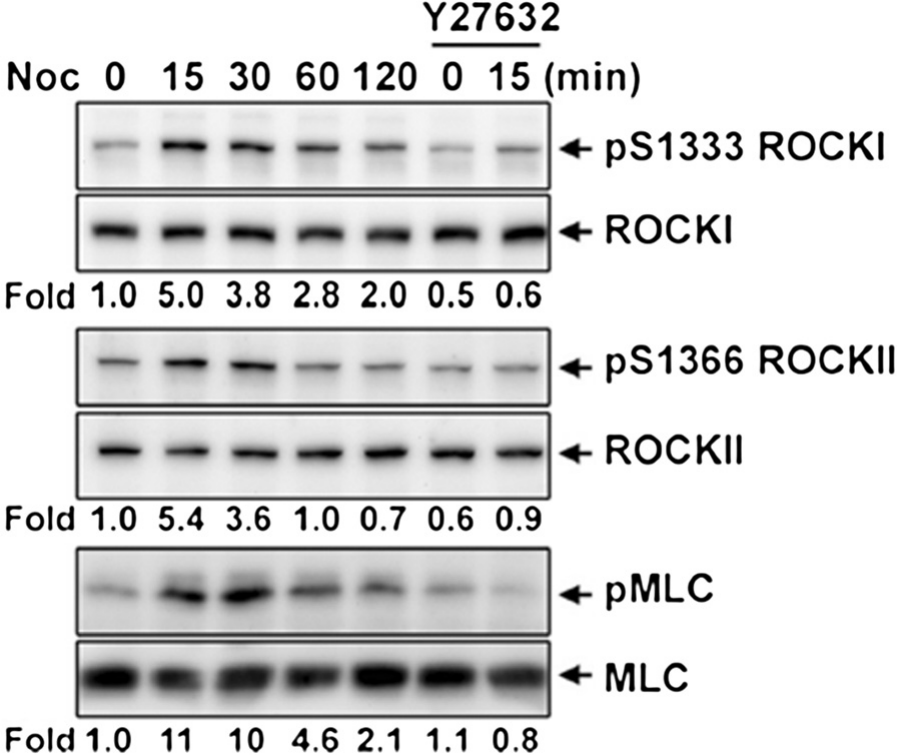
**Probing ROCKI and ROCKII activation in cells.** Normal mouse embryonic fibroblasts (MEFs) were serum starved for 24 hr and then stimulated with 10 μM of nocodazole in the presence or absence of 10 μM of Y27632. Cells were harvested at the indicated time for Western blotting with antibodies as indicated. The fold of relative protein phosphorylation was measured and showed.

We also examined the changes of ROCKI S1333 phosphorylation in serum-starved mouse embryonic fibroblasts (MEFs) that were treated with nocodazole to activate the RhoA signal via GEF-H1 released from microtubules disassembly [27]. As shown in Figure 4, ROCKI Ser1333 phosphorylation level was dramatically increased (5-fold) by nocodazole treatment at 15 min as seen in the increase of MLC phosphorylation. Similarly, the phosphorylation of ROCKII at Ser1366 was also increased. Treatment of cells with Y27632 prevented both nocodazole-induced ROCKI S1333 and ROCKII S1366 phosphorylation, suggesting the increase of the phosphorylation signal correlates with their corresponding kinase activation. The Y27632 treatment did not completely abolish the basal levels of ROCKI S1333 phosphorylation and ROCKII S1366 phosphorylation. It is uncertain whether this is due to the incomplete inhibition of ROCK kinase or a background level of non-specific signal. Nevertheless, the stimulation effect is reflected by these phosphorylation marks. Interestingly, the level of ROCKII S1366 phosphorylation was declined to the basal level at 60 min, while ROCKI S1333 phosphorylation sustained to 120 min. Thus, probing ROCKI Ser1333 and ROCKII Ser1366 phosphorylation is able to discriminate the differences in the kinetic of ROCKI and ROCKII activation in cells.

It is known that ROCKs form dimer [28-32]. In this study, we did not examine whether ROCKI S1333 phosphorylation is due to ROCKII in the heterodimer. It has been reported that the dimer consisting of wild-type kinase and catalytic-dead ROCK kinase domain is catalytically inactive [30]. Therefore, we are unable to test whether ROCKII can phosphorylate catalytic-dead ROCKI at S1333 in the heterodimer form. In an overexpression experiment, we found that the amount of homodimer of ROCKII was more than 10-fold higher than that of ROCKI/II (data not shown). Considering that all ROCKs purified from a variety of tissues have been shown to be in homodimer form [2,33-35], the physiological significance of S1333 phosphorylation in the heterodimer form of ROCKI/II is probably negligible. We also cannot exclude the possibility that other kinase is able to phosphorylate the S1333 site. Nevertheless, the correlation of S1333 phosphorylation with its upstream RhoA regulation and the extent of downstream substrate MLC phosphorylation suggest this modification as an indicator of ROCKI activation.

Small-molecule inhibitors against ROCK, such as Y27632 and Fasudil, have been developed to have potential in clinical implication [17,36,37]. Increasing number of clinical trials and animal experiments using these inhibitors suggest that ROCK activation plays an important role in the pathogenesis of many cardiovascular diseases, neurological disorders and cancers [3,4,38-43]. Although the functions of ROCKI and ROCKII are analogous and compensatory, genetic deletion studies suggest that each kinase might play distinct roles depending on tissue types and certain biological processes. Also unknown is which ROCK isoform is responsible for pathogenesis of a specific tissue in diseases and related to disease progression. Our antibodies that can detect the phosphorylation of ROCKI at Ser1333 and ROCKII at Ser1366 offer new opportunities to differentiate the activation status of ROCKI and II in association with diseases. Of note, all the current inhibitors cannot discern between ROCKI and II. The antibodies that can detect active forms of ROCKI and II provide valuable tools for screening ROCKI and ROCKII specific inhibitors.

## Conclusion

Ser1333 phosphorylation can indicate the active status of ROCKI in response to RhoA signaling. Thus, antibodies that recognize phosphorylation at Ser1333 and S1366 residues of ROCKI and II, respectively, are capable of probing their corresponding activation in biological samples. Also, these antibodies might be very useful reagents for drug screening of inhibitors specific against ROCKI and ROCKII isoform.

## Competing interests

The authors declare that they have no competing interests.

## Authors' contributions

HHC and SWL performed experiments. HHL and ZFC designed the study and wrote the paper. All authors read and approved the final paper.
